# Supplementary material for: Pattern recognition in reciprocal space with a magnon-scattering reservoir
Source: Nat Commun. 2023 Jul 4;14:3954. doi: 10.1038/s41467-023-39452-y (PMC10319722; doi:10.1038/s41467-023-39452-y)
Supplement: Supplementary file 1 — Supplementary Information [file 41467_2023_39452_MOESM1_ESM.pdf]

Supplementary Information for

Pattern recognition in reciprocal space with a magnon-scattering reservoir

Lukas Körber[1,2*†], Christopher Heins[1,2†], Tobias Hula[1,3], Joo-Von Kim[4], Sonia Thlang[4], Helmut Schultheiss[1,2], Jürgen Fassbender[1,2], Katrin Schultheiss[1*]

[1]Helmholtz-Zentrum Dresden - Rossendorf, Institut für Ionenstrahlphysik und Materialforschung, D-01328 Dresden, Germany
[2]Fakultät Physik, Technische Universität Dresden, D-01062 Dresden, Germany
[3]Institut für Physik, Technische Universität Chemnitz, 09107 Chemnitz, Germany
[4]Centre de Nanosciences et de Nanotechnologies, CNRS, Université Paris-Saclay, 91120 Palaiseau, France

[*]Correspondence to: l.koerber@hzdr.de, k.schultheiss@hzdr.de
[†]These authors contributed equally to this work.

This PDF file includes:

Supplementary Notes 1 and 2
Supplementary Figures 1 to 8

Supplementary Note 1: 4-symbol pulse pattern recognition from simulations. The output space for the pattern recognition task was constructed from the power spectral density using a binning technique, as discussed in the main text, where the power in curves such as Supplementary Fig. 3d are integrated over frequency intervals of different sizes. For the scattered modes, bins are constructed within a 4-GHz frequency interval running from 2.4 to 6.4 GHz (except for the case where $f_A = 8.9$ GHz and $f_D = 10.7$ GHz, where the interval is 3.0 to 7.0 GHz). With a frequency resolution of 12.5 MHz, which is determined from the total duration of the simulation run (80 ns), the 4-GHz interval is binned using 4, 5, 8, 10, 16, or 20 spectral points, resulting in bin sizes of 50, 62.5, 100, 125, and 200 MHz, respectively. This results in an output state vector $\boldsymbol{Y}$ of dimension 80, 64, 40, 32, 20, and 16, respectively. When we consider an output space based on the directly-excited modes for purposes of comparison, we use two frequency bins, centered at f_A and f_B, respectively, with the same range of sizes as above. In this case, the dimension of the output vector is always two.

For a given set of inputs, (f_A, f_B) and $(b_{\mathrm{rf},A}, b_{\mathrm{rf},B})$, we performed 200 simulations for each 4-symbol pulse pattern, i.e., a total of 3200 simulations were executed. For each of these simulations, a different seed for the random number generator was used so that the realization of the thermal field $\boldsymbol{b}_{\mathrm{th}}$ differs from one simulation to the next. Of the 200 simulations for each 4-symbol pulse pattern, we tested different sizes of the subsets allocated to training, validation, and testing set. Some examples are shown in Supplementary Figs. 4a-c.

For a given subset split, we performed supervised learning using the `Classify` function[1] in MATHEMATICA (v13.0.1) with the training, validation, and testing data to determine the accuracy of the pattern recognition task. In order to reduce bias in the trained models, we performed training on five different permutations of the training, validation, and testing sets drawn from the same 200 simulations of a given 4-pulse AB pattern, and averaged over these results. For instance, 100% recognition rates in the confusion matrices correspond to 100 correct predictions out of 100 attempts based on five test sets of 20 simulations.

Supplementary Figs. 4a-c show the performance of the logistic regression algorithm on the three subset splits, with the (80% training / 10% validation / 10% testing) case giving the best performance, although the qualitative features between the three cases are very similar. With this split, we also compared the performance with other supervised learning algorithms, such as Nearest Neighbors, Random Forest, and Support Vector Machine, and found similar results. In all cases, the output space constructed from the directly-excited modes leads to poorer performance in comparison with that using the scattered modes, with the only exception involving the Nearest Neighbors algorithm. The main text features the (80% / 10% / 10%) split with the logistic regression algorithm as this is representative of the performance of the MSR.

Confusion matrices for three input combinations shown in Fig. 4b of the main text, i.e., 6.5 GHz and 7.4 GHz (3.0 mT), 6.5 and 8.9 GHz (3.0 mT), and 8.9 and 10.7 GHz (3.0 mT), with a frequency bin of 100 MHz, are given in Supplementary Fig. 5.

As for the primary case considered in the main text [7.4 GHz (3.5 mT) and 8.9 GHz (3.0 mT)], we can observe significant scatter across the different 4-symbol pulse patterns for the directly-excited modes, while the learning based on the scattered modes results in good recognition rates

[1]Wolfram Research (2014), Classify, Wolfram Language function, https://reference.wolfram.com/language/ref/Classify.html (updated 2021).

across the board. A comparison of the confusion matrices for different fluctuation amplitudes with the primary case, which complement the data in Figs. 4d and 4e, is shown in Supplementary Fig. 6.

Supplementary Note 2: Extension of the input space. In order to showcase the feasibility of more complex input patterns, we recorded the spectral response to different combinations of 3- and 4-symbol sequences. Supplementary Fig. 7 shows the simulated power spectral density of the scattered spin wave modes for the different permutations of the symbols 'A', 'B', 'C', and 'D' in a 3- and 4-symbol sequence. The excitation frequency and power was chosen in accordance with the main text. In addition, for the three symbols 'A', 'B', and 'C' the time-resolved spectral response was experimentally recorded by TR-μBLS and is shown in Supplementary Fig. 8. As it can be seen in both experiment and simulation, the spectra are qualitatively different for the varying input sequences.

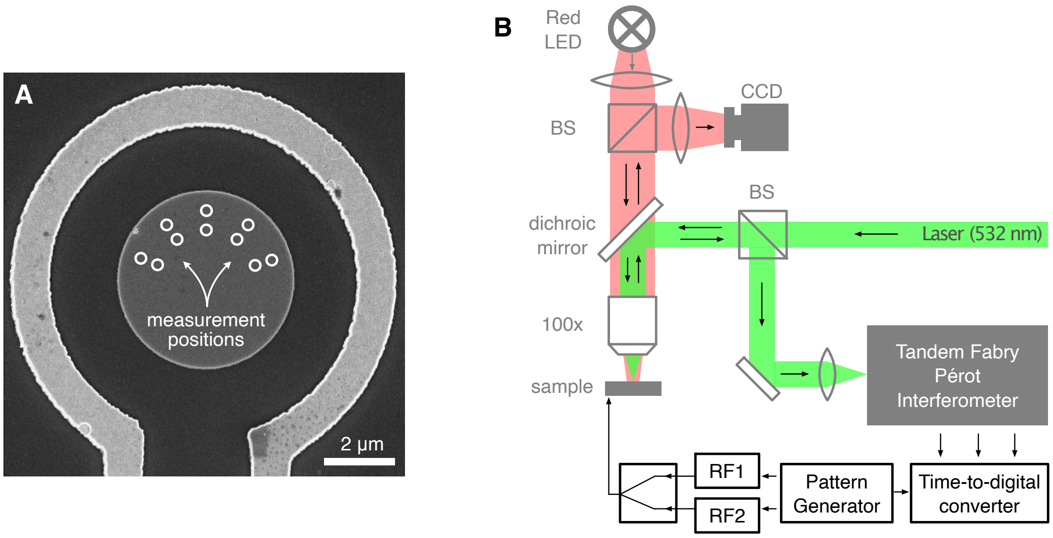

Supplementary Figure 1: Sample preparation and characterization. **a** Scanning-electron-microscopy image of the magnetic disk used as a magnon-scattering reservoir. Hallow circles mark the positions of the microfocused laser spot in the Brillouin-light-scattering-microscopy experiments. **b** Schematics of the experimental setup, including the Brillouin-light-scattering optics and the microwave generators to generate the input pulses.

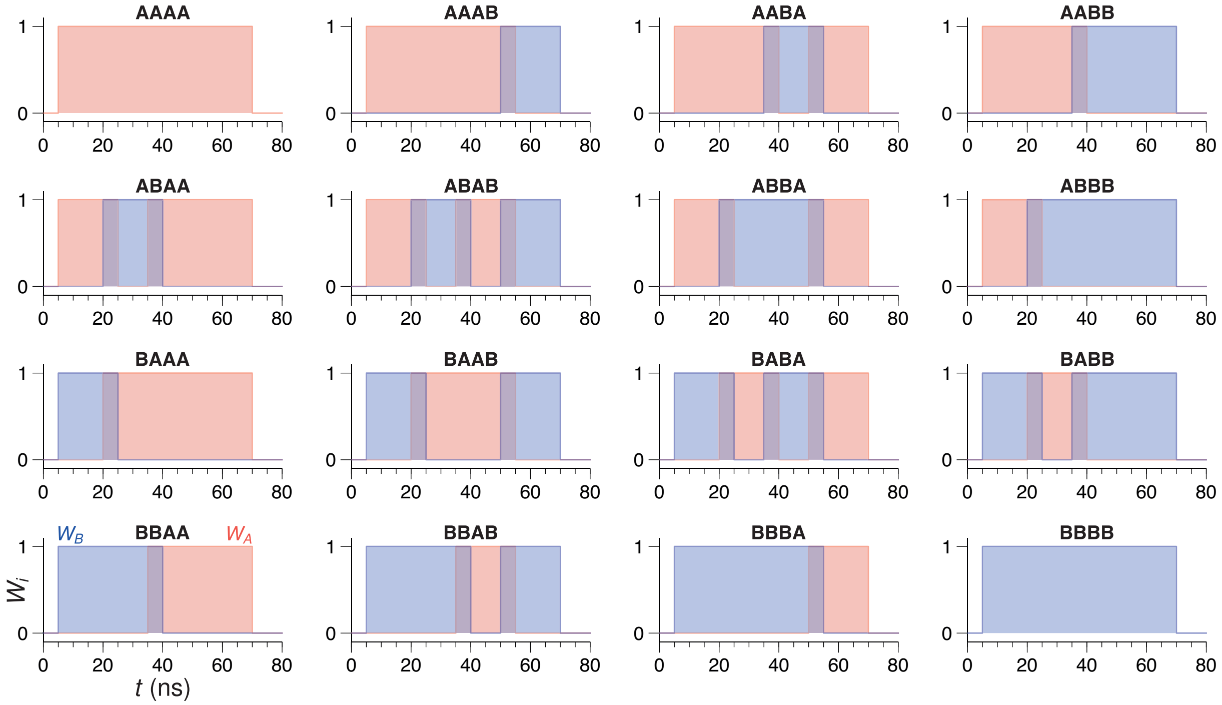

Supplementary Figure 2: Excitation with oscillating fields. Windowing functions $W_i(t)$ for the "A" and "B" patterns encoded in the excitation field $\boldsymbol{b}_{\rm rf}(t)$.

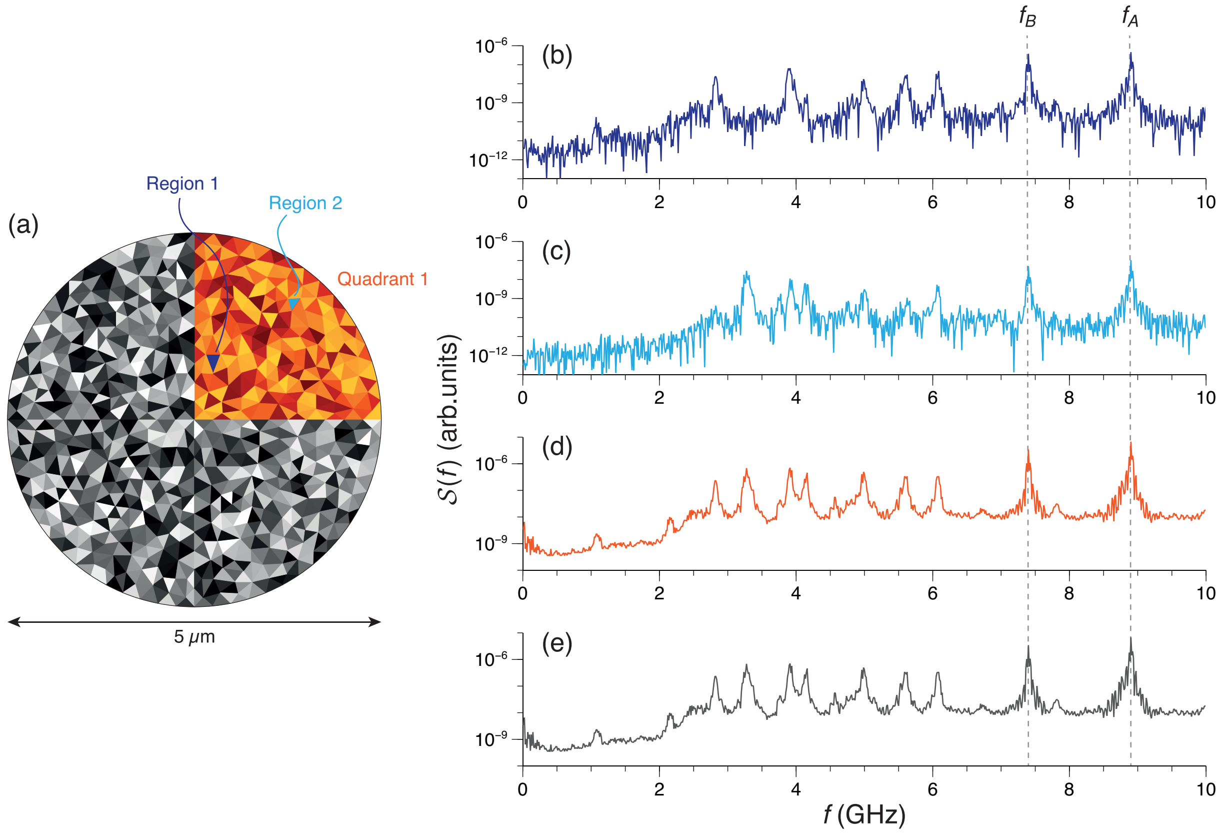

Supplementary Figure 3: Calculating the power spectral density of spin wave excitations using a coarse-graining procedure. **a** Triangle mesh of the simulation geometry. (**b–e**) Power spectral density, $\mathcal{S}(f)$, as a function of the frequency, f, for magnetization excitations in **b** Region 1, **c** Region 2, **d** Quadrant 1, and **e** the entire disk. The excitations are driven by an ABAB sequence with $\mathrm{f_A} = 8.9\,\mathrm{GHz}$ and $\mathrm{f_B} = 7.4\,\mathrm{GHz}$, and with $\mathrm{b_{rf,A}} = 3.0\,\mathrm{mT}$ and $\mathrm{b_{rf,B}} = 3.5\,\mathrm{mT}$.

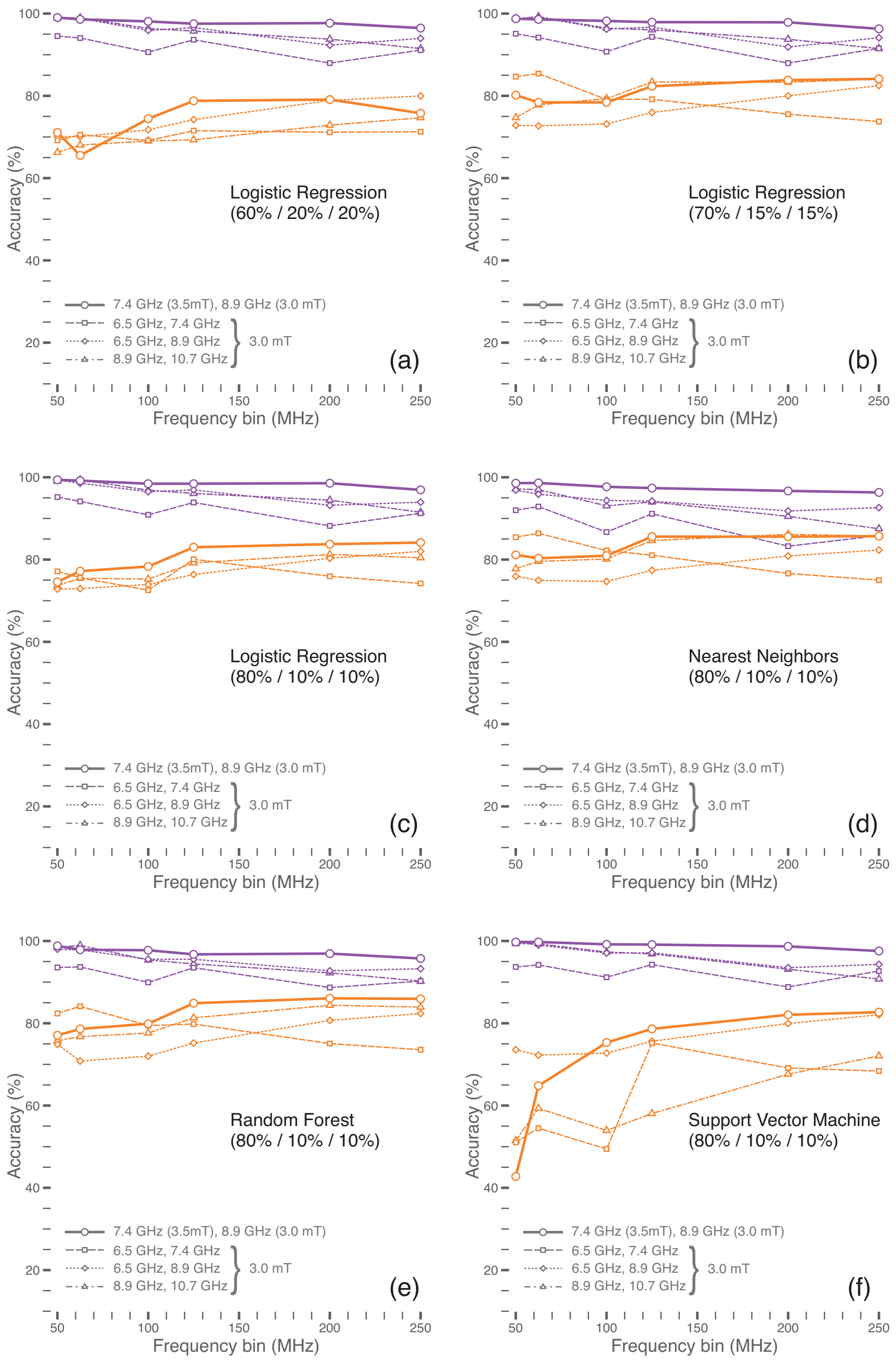

Supplementary Figure 4: Comparison of different subset sizes for the training, validation, and testing data (given as percentages), along with different supervised learning algorithms used. (**a**) Logistic regression with 60% of the dataset for training, 20% for validation, and 20% for testing (60% / 20% / 20%). (**b**) Logistic regression with a split of (70% / 15% / 15%). (**c**) Logistic regression with a split of (80% / 10% / 10%). (**d**) Nearest neighbors algorithm with a split of (80% / 10% / 10%). (**e**) Random Forest algorithm with a split of (80% / 10% / 10%). (**f**) Support Vector Machine algorithm with a split of (80% / 10% / 10%).

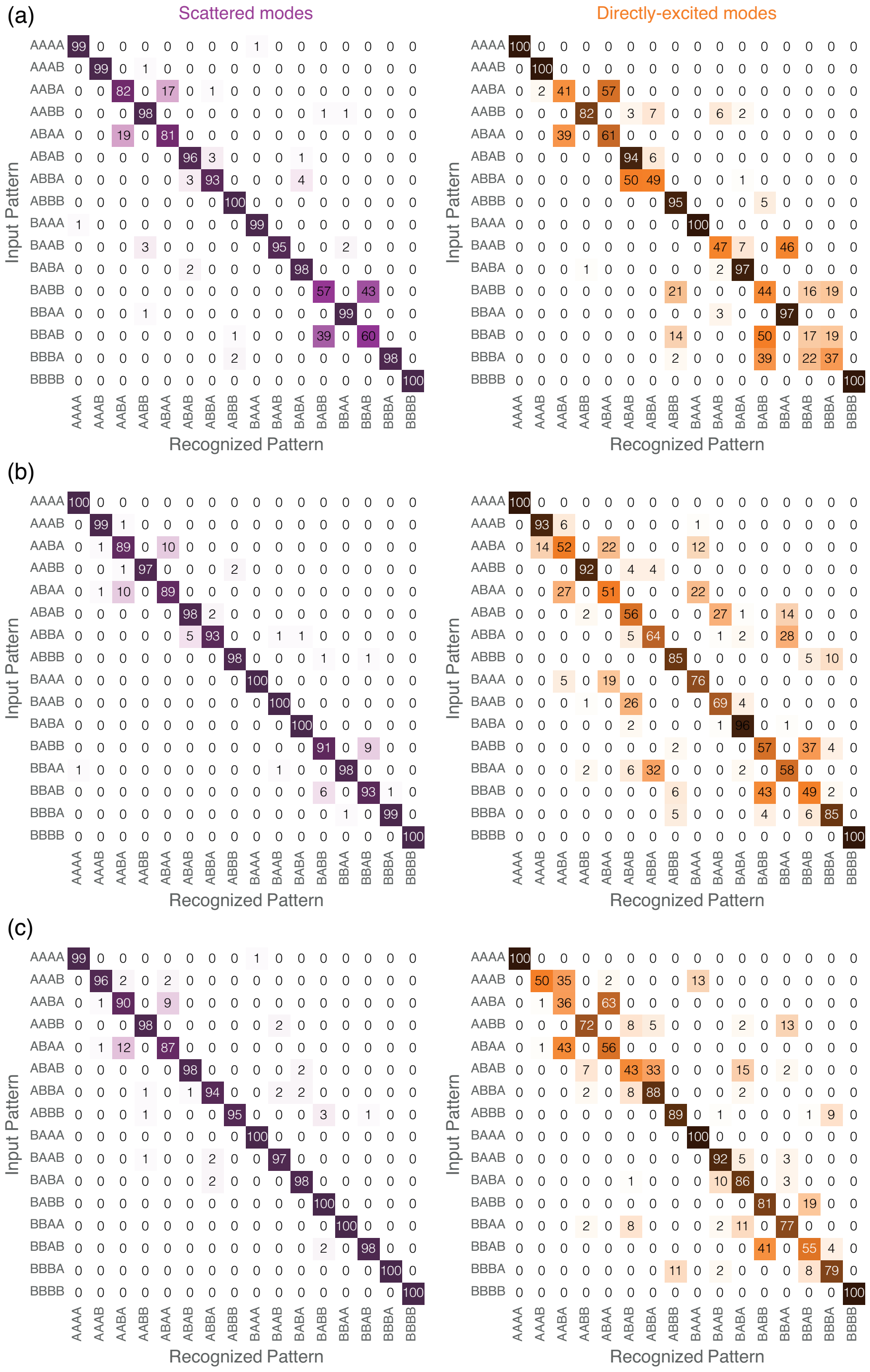

Supplementary Figure 5: Confusion matrices for output spaces based on scattered modes and directly-excited modes with a frequency bin of 100 MHz and input parameters of (**a**) 6.5 GHz and 7.4 GHz (3.0 mT), (**b**) 6.5 and 8.9 GHz (3.0 mT), (**c**) 8.9 and 10.7 GHz (3.0 mT).

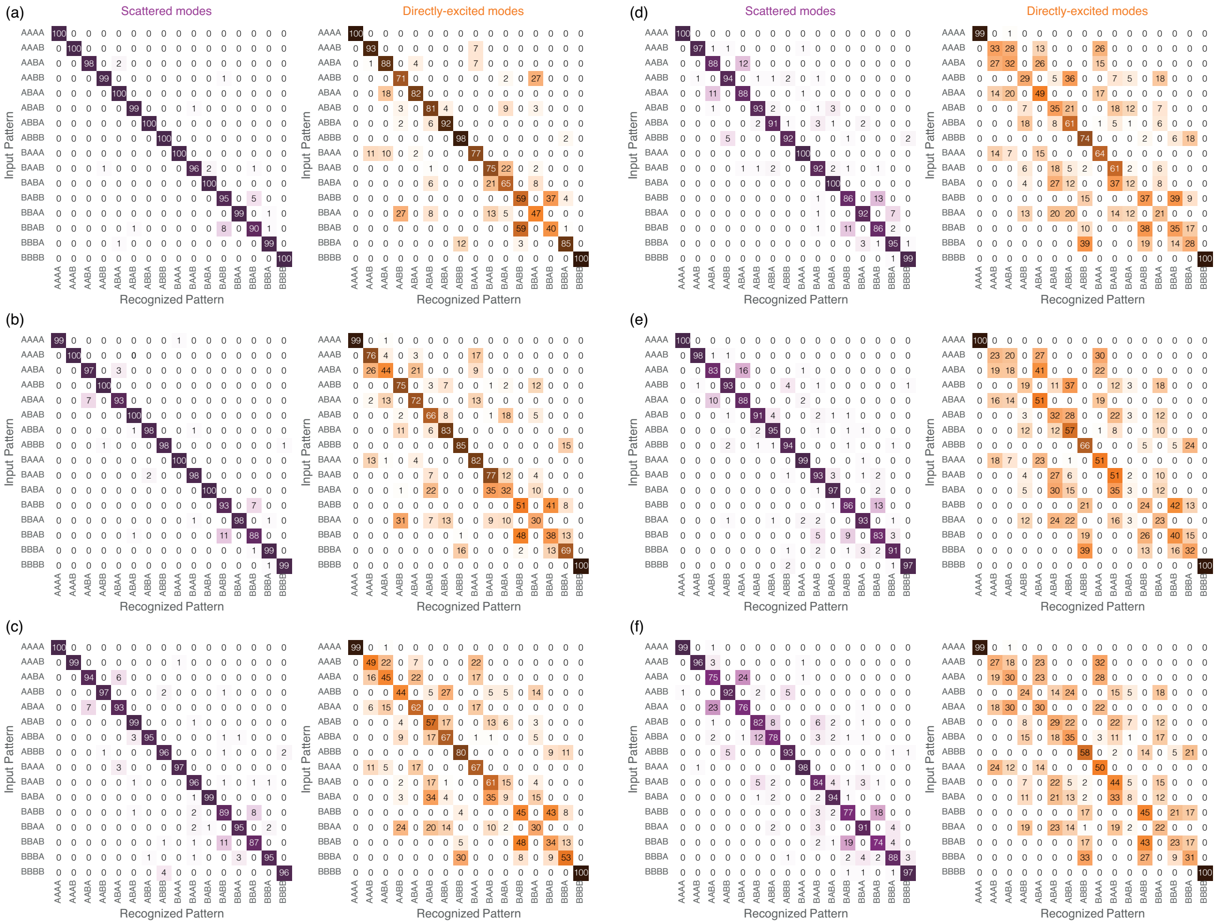

Supplementary Figure 6: Confusion matrices for output spaces based on scattered modes and directly-excited modes with a frequency bin of 100 MHz and input parameters of 7.4 GHz (3.5 mT) and 8.9 GHz (3.0 mT), with different levels of noise in the rf field amplitudes: (**a**) 0%, (**b**) 2%, (**c**) 4%, (**d**) 6%, (**e**) 8%, (**f**) 10%.

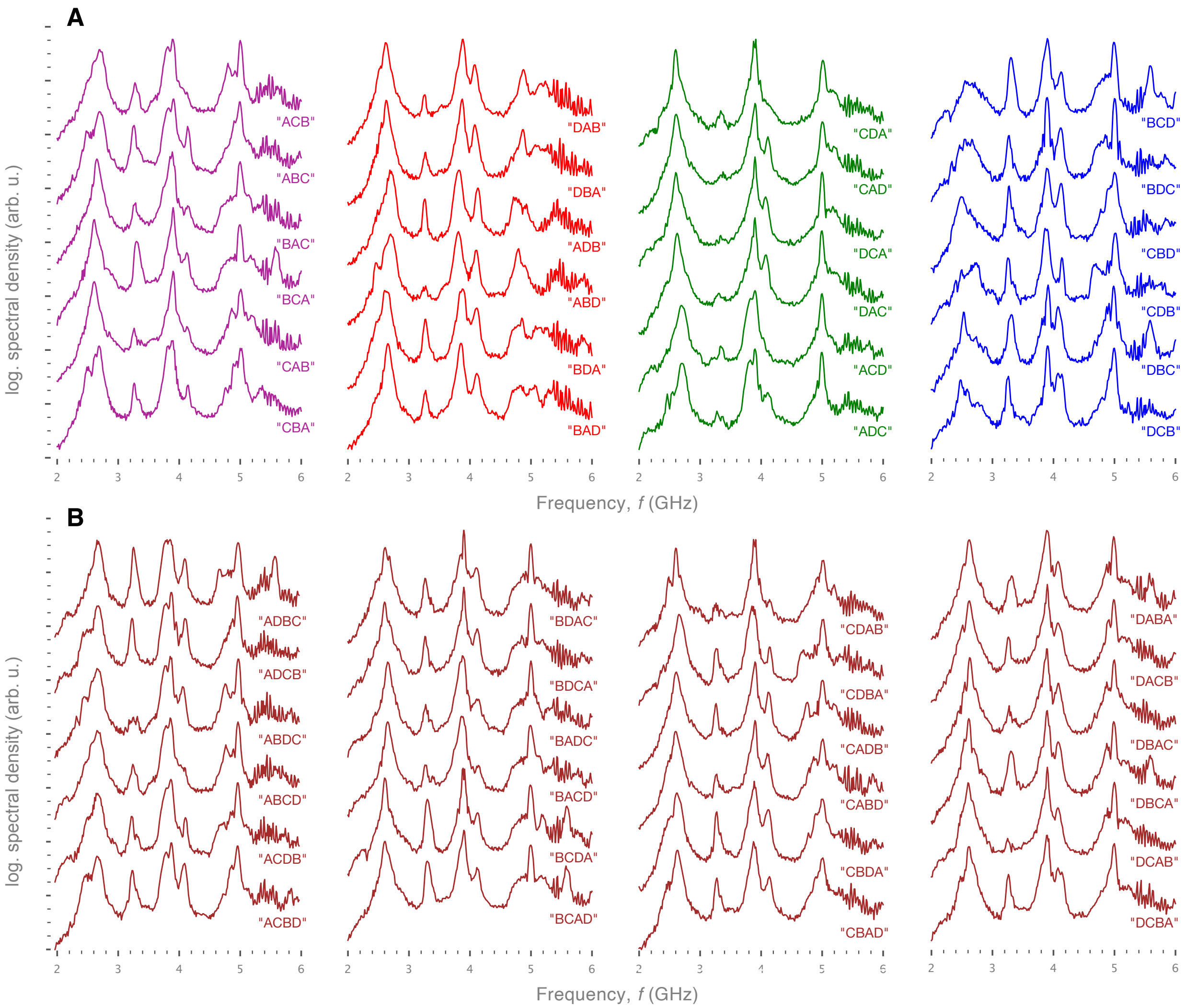

Supplementary Figure 7: Simulated power spectral density (PSD) of the scattered modes for **a** 3-symbol and **b** 4-symbol input sequences, where $f_A = 8.9\,\text{GHz}$ (3.0 mT), $f_B = 7.4\,\text{GHz}$ (3.5 mT), $f_C = 6.5\,\text{GHz}$ (3.0 mT), and $f_D = 10.7\,\text{GHz}$ (3.0 mT), as considered in the main text.

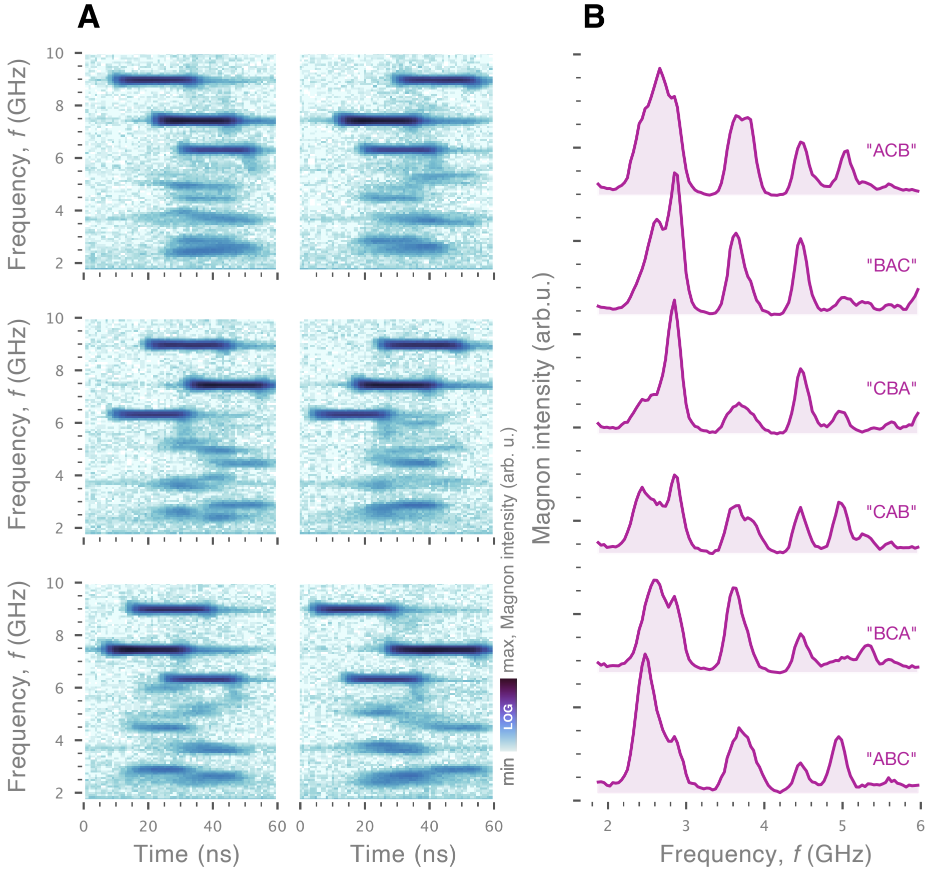

Supplementary Figure 8: **a** Time-resolved spectral response of the MSR to a three-symbol microwave pattern "ABC", detected with TR-μBLS, where $f_A = 8.9\,\mathrm{GHz}$ (25 dBm), $f_B = 7.4\,\mathrm{GHz}$ (23 dBm), $f_C = 6.3\,\mathrm{GHz}$ (20 dBm). The input consists of 3 pulses with a duration of 20 ns and an overlap of 10 ns. **b** Time averaged spectra of the MSR in response to different permutations of the three input frequencies.
